# Effect of prone positioning on oxygenation and static respiratory system compliance in COVID-19 ARDS vs. non-COVID ARDS

**DOI:** 10.1186/s12931-021-01819-4

**Published:** 2021-08-06

**Authors:** Jimyung Park, Hong Yeul Lee, Jinwoo Lee, Sang-Min Lee

**Affiliations:** Division of Pulmonary and Critical Care Medicine, Department of Internal Medicine, Seoul National University Hospital, Seoul National University College of Medicine, 101, Daehak-ro, Jongno-gu, Seoul, 03080 Republic of Korea

**Keywords:** COVID-19, Acute respiratory distress syndrome, Prone position, Oxygenation, Respiratory system compliance

## Abstract

**Background:**

Prone positioning is recommended for patients with moderate-to-severe acute respiratory distress syndrome (ARDS) receiving mechanical ventilation. While the debate continues as to whether COVID-19 ARDS is clinically different from non-COVID ARDS, there is little data on whether the physiological effects of prone positioning differ between the two conditions. We aimed to compare the physiological effect of prone positioning between patients with COVID-19 ARDS and those with non-COVID ARDS.

**Methods:**

We retrospectively compared 23 patients with COVID-19 ARDS and 145 patients with non-COVID ARDS treated using prone positioning while on mechanical ventilation. Changes in PaO_2_/FiO_2_ ratio and static respiratory system compliance (Crs) after the first session of prone positioning were compared between the two groups: first, using all patients with non-COVID ARDS, and second, using subgroups of patients with non-COVID ARDS matched 1:1 with patients with COVID-19 ARDS for baseline PaO_2_/FiO_2_ ratio and static Crs. We also evaluated whether the response to the first prone positioning session was associated with the clinical outcome.

**Results:**

When compared with the entire group of patients with non-COVID ARDS, patients with COVID-19 ARDS showed more pronounced improvement in PaO_2_/FiO_2_ ratio [adjusted difference 39.3 (95% CI 5.2–73.5) mmHg] and static Crs [adjusted difference 3.4 (95% CI 1.1–5.6) mL/cmH_2_O]. However, these between-group differences were not significant when the matched samples (either PaO_2_/FiO_2_-matched or compliance-matched) were analyzed. Patients who successfully discontinued mechanical ventilation showed more remarkable improvement in PaO_2_/FiO_2_ ratio [median 112 (IQR 85–144) vs. 35 (IQR 6–52) mmHg, *P* = 0.003] and static compliance [median 5.7 (IQR 3.3–7.7) vs. − 1.0 (IQR − 3.7–3.0) mL/cmH_2_O, *P* = 0.006] after prone positioning compared with patients who did not. The association between oxygenation and Crs responses to prone positioning and clinical outcome was also evident in the adjusted competing risk regression.

**Conclusions:**

In patients with COVID-19 ARDS, prone positioning was as effective in improving respiratory physiology as in patients with non-COVID ARDS. Thus, it should be actively considered as a therapeutic option. The physiological response to the first session of prone positioning was predictive of the clinical outcome of patients with COVID-19 ARDS.

**Supplementary Information:**

The online version contains supplementary material available at 10.1186/s12931-021-01819-4.

## Background

After its first outbreak in Wuhan, China in December 2019, coronavirus disease 2019 (COVID-19) spread rapidly around the world and continues to be a global threat [1]. Although most patients with COVID-19 have mild manifestations, the condition deteriorates in approximately 10–20% of patients, requiring admission to an intensive care unit and invasive mechanical ventilation for acute respiratory distress syndrome (ARDS) [2–4]. Whether ARDS due to COVID-19 (COVID-19 ARDS) is clinically distinct from ARDS due to other causes (non-COVID ARDS) has been a controversial issue [5, 6].

Prone positioning is currently implemented for patients with moderate-to-severe ARDS with the potential to reduce mortality [7]. The beneficial effect of prone positioning on oxygenation has been known for decades, but whether the improvement in oxygenation is directly associated with patients’ survival gain has been questionable [8]. We have recently shown that the extent of improvement in the ratio of partial pressure of arterial oxygen (PaO_2_) to the fraction of inspired oxygen (FiO_2_) after prone positioning could be a predictor of survival of patients with ARDS [9].

In this study, we aimed to investigate whether physiological responses to prone positioning differ between patients with COVID-19 ARDS and those with non-COVID ARDS, focusing not only on oxygenation, but also on static respiratory system compliance (Crs), considering recent studies that reported a prognostic value of static Crs for COVID-19 ARDS [10, 11]. We also evaluated whether the response to the first session of prone positioning was associated with patients’ clinical outcome.

## Methods

### Patients with COVID-19 ARDS

This study was a retrospective cohort study using the patients’ medical records conducted at the Seoul National University Hospital, a tertiary referral hospital in South Korea, which has served as a nationally designated hospital for patients with severe and critical COVID-19. This study was approved by the institutional review board of the Seoul National University Hospital (IRB No. 2012-036-1179). We reviewed the records of all patients older than 18 years who were admitted to our center between January and December 2020 after being diagnosed as having COVID-19 using reverse transcription-polymerase chain reaction assay. Among such patients, those for whom mechanical ventilation was initiated and prone positioning was implemented were included in this study.

### Treatment and prone positioning

Patients with COVID-19 ARDS were treated based on the most updated evidence at the time of their hospitalization [12, 13]. In patients with worsening respiratory failure, we usually used a high flow nasal cannula at first, but mechanical ventilation with endotracheal intubation was initiated in refractory cases [14]. If PaO_2_/FiO_2_ ratio after initiation of mechanical ventilation was less than 200 mmHg, we actively considered prone positioning with neuromuscular blockade [7, 15]. Prone position was maintained for at least 16 h per day [7]. Discontinuation of prone positioning was considered if reduction in ventilator assistance was possible allowing for spontaneous or assisted ventilation.

### Comparison with non-COVID ARDS

We reviewed every patient with non-COVID ARDS treated using prone positioning while on mechanical ventilation since January 2014 until December 2020, and the cohort of these patients was used for a comparison between COVID-19 ARDS and non-COVID ARDS. Some of these patients were included in our previous study [9]. First, we used the entire group of patients with non-COVID ARDS while adjusting for the between-group differences. Second, for a more accurate comparison, patients with COVID-19 ARDS were matched with subgroup populations among the non-COVID ARDS group: one matched 1:1 for PaO_2_/FiO_2_ ratio and one matched 1:1 for static Crs.

### Study outcome and data collection

The primary outcome of this study was the extent of changes in PaO_2_/FiO_2_ ratio and static Crs after the first prone positioning session. In each patient, the changes in PaO_2_/FiO_2_ ratio and static Crs were tracked during the first prone positioning session. Using the results of arterial blood gas analysis and the ventilator setting at the time of blood sampling, PaO_2_/FiO_2_ ratio and static Crs were evaluated at four timepoints for each patient: baseline (before initiation of prone positioning), P1 (approximately 10 h after initiation of prone positioning), P2 (approximately 16 h after initiation of prone positioning, which is the last timepoint before cessation of prone positioning), and S1 (approximately 2 h after changing to supine position). For the main outcome of this study, each patient’s response to the first session of prone positioning was calculated as the difference in PaO_2_/FiO_2_ ratio and static Crs between the baseline and P2 timepoints. In addition, we aimed to evaluate whether the physiological responses to prone positioning correlate with the clinical outcomes of patients, given the controversial results of previous studies [9, 16].

### Statistical analysis

We assessed the differences between patients with COVID-19 ARDS and those with non-COVID ARDS and *P* values of < 0.05 for two-tailed tests were considered statistically significant. First, all patients with non-COVID ARDS were compared with patients with COVID-19 ARDS. Then, two subgroup populations of patients with non-COVID ARDS were used for 1:1 matched comparison with patients with COVID-19 ARDS (PaO_2_/FiO_2_-matched subgroup and compliance-matched subgroup). The matching was performed using an optimal algorithm without replacement [17].

For each patient, the Wilcoxon singed-rank test was used to compare the PaO_2_/FiO_2_ ratio and static Crs between different timepoints Then, the extent of changes in these parameters from baseline to P2 timepoints was compared between the COVID-19 ARDS and non-COVID ARDS groups using multivariable linear regression analysis. Comparisons between the matched samples were performed similarly [18]. Because there are no definite well-known predictors for response to prone positioning, we adjusted for age, sex, body mass index, duration of mechanical ventilation before the initiation of prone positioning, sequential organ failure assessment (SOFA) score, Charlson comorbidity index (CCI), and baseline setting of mechanical ventilator (positive end-expiratory pressure [PEEP] and tidal volume) as well as baseline PaO_2_/FiO_2_ ratio, static Crs, and ventilatory ratio. Ventilatory ratio was selected as a parameter to assess the efficacy of ventilation because we did not routinely monitor the expired CO_2_ level [19].

For patients with COVID-19 ARDS treated using prone positioning, we assessed whether the response of PaO_2_/FiO_2_ and static Crs could predict patients’ probability of successful discontinuation of mechanical ventilation within 90 days using a receiver operating characteristic (ROC) analysis. In addition, the Fine and Gray competing risk regression analysis was performed to calculate the subdistribution hazard ratio (SHR) and 95% confidence interval (CI) with adjustment for age, sex, SOFA score, CCI, and baseline PaO_2_/FiO_2_ ratio and static Crs [20, 21]. Death occurring during mechanical ventilation was considered as the competing event. Patients who were still dependent on mechanical ventilation were censored at 90 days after the first prone positioning session. All statistical analyses were performed using STATA software (version 14.0; StataCorp LP, College Station, TX, USA).

## Results

### Clinical characteristics of patients

Until December 2020, 46 patients with COVID-19 ARDS were treated at our center using mechanical ventilation. Among them, 23 patients (50%) did not start prone positioning because their oxygenation status rapidly improved after initiation of mechanical ventilation. The remaining 23 patients (50%) were treated using prone positioning for persistent moderate-to-severe ARDS. The median interval between the diagnosis of COVID-19 and initiation of prone positioning was 9 (interquartile range [IQR] 4–12) days. To compare with patients with COVID-19 ARDS, 145 patients with non-COVID ARDS treated using prone positioning were reviewed and among them, two subgroups of 23 patients (1:1 matched for PaO_2_/FiO_2_ ratio and static Crs, respectively) were selected.

Comparison of baseline characteristics and respiratory mechanics between these groups are described in Tables 1, 2. The patients with non-COVID ARDS had more comorbidities and they were more severely ill with more organ dysfunctions and higher SOFA scores than the patients with COVID-19 ARDS. They also showed worse oxygenation (median PaO_2_/FiO_2_ ratio 96 vs. 107 mmHg, *P* = 0.037) and lower static Crs (median 21.9 vs. 27.2 mL/cmH_2_O, *P* = 0.005). All patients in both groups received ventilation with low tidal volume, but patients with non-COVID ARDS had higher ventilatory ratio (median 2.2 vs. 1.7, *P* < 0.001), requiring higher minute ventilation (median 177 vs. 140 mL/kg/min, *P* < 0.001). Among the patients with non-COVID ARDS, 1:1 matching was well performed, showing no between-group differences in the median values of PaO_2_/FiO_2_ ratio and static Crs in PaO_2_/FiO_2_-matched and compliance-matched samples, respectively.

**Table 1:** Patient characteristics

Variables	COVID-19 ARDS	Non-COVID ARDS					
	Entire group N = 23	Entire group N = 145	*P* value^a^	PaO_2_/FiO_2_-matched N = 23	*P* value^a^	Compliance-matched N = 23	*P* value^a^
Age, years	70 (63–74)	67 (59–74)	0.222	75 (70–79)	0.092	66 (60–74)	0.159
Male sex	15 (65.2%)	97 (66.9%)	0.874	17 (73.9%)	0.522	16 (69.6%)	0.753
Height, cm	165 ± 9	163 ± 8	0.374	163 ± 10	0.614	164 ± 8	0.791
Body weight, kg	70 (58–79)	61 (53–70)	0.018	57 (51–69)	0.015	64 (55–69)	0.132
Body mass index, kg/m^2^	25.6 (22.9–27.4)	22.9 (20.8–26.0)	0.024	22.5 (19.3–24.7)	0.009	22.7 (20.5–27.2)	0.121
Interval between intubation and the first prone positioning session, days	1 (0–2)	2 (1–5)	0.009	1 (0–3)	0.116	2 (1–4)	0.047
Total number of sessions of prone positioning	4 (3–9)	2 (1–4)	< 0.001	2 (1–4)	0.008	2 (1–4)	0.011
Mean duration of prone positioning per session, hours	18 (17–19)	18 (16–19)	0.653	17 (16–18)	0.180	17 (16–20)	0.231
Charlson comorbidity index	4 (3–4)	5 (3–8)	0.012	5 (4–6)	0.011	5 (4–7)	0.032
APACHE II score	20 (12–25)	29 (25–33)	< 0.001	30 (26–35)	< 0.001	29 (25–37)	< 0.001
SAPS II score	42 (31–61)	65 (55–71)	< 0.001	66 (62–78)	< 0.001	65 (58–78)	< 0.001
SOFA score	8 (5–11)	12 (9–14)	< 0.001	12 (9–14)	0.002	13 (11–15)	< 0.001

*APACHE* acute physiology and chronic health evaluation, *ARDS* acute respiratory distress syndrome, *SAPS* Simplified Acute Physiology Score, *SOFA* sequential organ failure assessment

^a^*P* values are for comparison between patients with COVID-19 ARDS and patients with non-COVID ARDS

**Table 2:** Baseline respiratory mechanics and clinical outcomes

Variables	COVID-19 ARDS	Non-COVID ARDS					
	Entire group N = 23	Entire group N = 145	*P* value^a^	PaO_2_/FiO_2_-matched N = 23	*P* value^a^	Compliance-matched N = 23	*P* value^a^
Arterial blood gas analysis							
pH	7.37 (7.34–7.39)	7.34 (7.28–7.40)	0.173	7.36 (7.26–7.40)	0.291	7.34 (7.28–7.41)	0.568
PaCO_2_, mmHg	44 (40–49)	49 (40–55)	0.139	48 (45–54)	0.071	46 (37–54)	0.860
PaO_2_, mmHg	75 (66–80)	71 (62–85)	0.467	79 (65–93)	0.391	69 (55–78)	0.097
HCO_3_, mEq/L	24.9 (24.1–28.4)	24.2 (21.5–27.3)	0.161	23.3 (21–28.6)	0.191	23.3 (18.6–28.6)	0.240
Ventilator FiO_2_	0.7 (0.6–0.8)	0.8 (0.65–1.0)	0.047	0.75 (0.6–0.9)	0.537	0.75 (0.7–1.0)	0.221
PaO_2_/FiO_2_ ratio, mmHg	107 (92–132)	96 (74–120)	0.037	107 (92–131)	1.000	90 (72–104)	0.007
PEEP, cmH_2_O	12 (9–13)	10 (8–11)	0.016	10 (7–10)	0.063	10 (8–11)	0.135
Driving pressure, cmH_2_O	13 (12–16)	18 (15–21)	< 0.001	18 (15–22)	0.001	15 (13–18)	0.096
Respiratory rate, breaths/min	21 (19–27)	27 (24–30)	0.002	26 (25–30)	0.012	24 (21–30)	0.116
Tidal volume per PBW, mL/kg	6.3 (5.6–7.0)	6.6 (6.0–7.3)	0.126	6.4 (5.9–7.2)	0.734	6.5 (5.9–9.0)	0.177
Minute ventilation per PBW, mL/kg/min	140 (123–171)	177 (145–200)	< 0.001	167 (133–193)	0.044	173 (141–194)	0.003
Static respiratory system compliance, mL/cmH_2_O	27.2 (21.9–32.7)	21.9 (18.2–26.5)	0.005	20.0 (15.6–27.2)	0.008	27.2 (21.9–32.7)	0.983
Ventilatory ratio	1.7 (1.4–2.0)	2.2 (1.7–2.7)	< 0.001	2.1 (1.7–2.5)	0.015	2.2 (1.7–2.6)	0.002
Laboratory results							
White blood cell, 10^3^/μL	10.46 (6.53–16.04)	13.36 (5.73–17.77)	0.717	15.73 (13.14–23.71)	0.008	10.01 (5.73–16.86)	0.904
Segmented neutrophil, 10^3^/μL	9.66 (5.88–14.57)	11.42 (4.93–15.06)	0.906	14.40 (11.53–19.56)	0.014	9.56 (4.53–14.43)	0.684
Lymphocyte, 10^3^/μL	0.69 (0.48–0.86)	0.43 (0.18–0.84)	0.047	0.70 (0.27–0.98)	0.895	0.51 (0.16–0.88)	0.249
C-reactive protein, mg/dL	10.9 (6.3–19.8)	13.5 (6.6–21.5)	0.234	13.8 (7.6–21.5)	0.449	19.2 (9.1–25.7)	0.037
RT-PCR for SARS-CoV-2							
Ct value for *env* gene	23.01 ± 4.99						
Ct value for *RdRp* gene	22.60 ± 5.22						
Adjunctive therapies							
Inhaled nitric oxide	8 (34.8%)	56 (38.6%)	0.725	4 (17.4%)	0.179	8 (34.8%)	1.000
Renal replacement therapy	4 (17.4%)	29 (20.0%)	1.000	2 (8.7%)	0.665	7 (30.4%)	0.491
ECMO or ECCO_2_R	3 (13.0%)	7 (4.8%)	0.141	0 (0.0%)	0.233	2 (8.7%)	1.000
Tracheostomy	12 (52.2%)	59 (40.7%)	0.300	15 (65.2%)	0.369	6 (26.1%)	0.070
90-days clinical outcome							
Successful discontinuation of mechanical ventilation	16 (69.6%)	27 (18.6%)	< 0.001	5 (21.7%)	0.005	4 (17.4%)	0.001
Dependent on mechanical ventilation	2 (8.7%)	11 (7.6%)		5 (21.7%)		1 (4.3%)	
Death	5 (21.7%)	107 (73.8%)		13 (56.6%)		18 (78.3%)	
Ventilator free days	45 (0–82)	0 (0–0)	< 0.001	0 (0–0)	0.002	0 (0–0)	0.002

*ARDS* acute respiratory distress syndrome, *Ct value* cycle threshold value, *ECCO*_*2*_*R* extracorporeal carbon dioxide removal, *ECMO* extracorporeal membrane oxygenation, *FiO*_*2*_ fraction of inspired oxygen, *PaCO*_*2*_ partial pressure of carbon dioxide, *PaO*_*2*_ partial pressure of oxygen, *PBW* predicted body weight, *PEEP* positive end expiratory pressure, *RT-PCR* reverse transcription polymerase chain reaction

^a^*P* values are for comparison between patients with COVID-19 ARDS and patients with non-COVID ARDS

### Oxygenation and static compliance responses

**Fig. 1:**
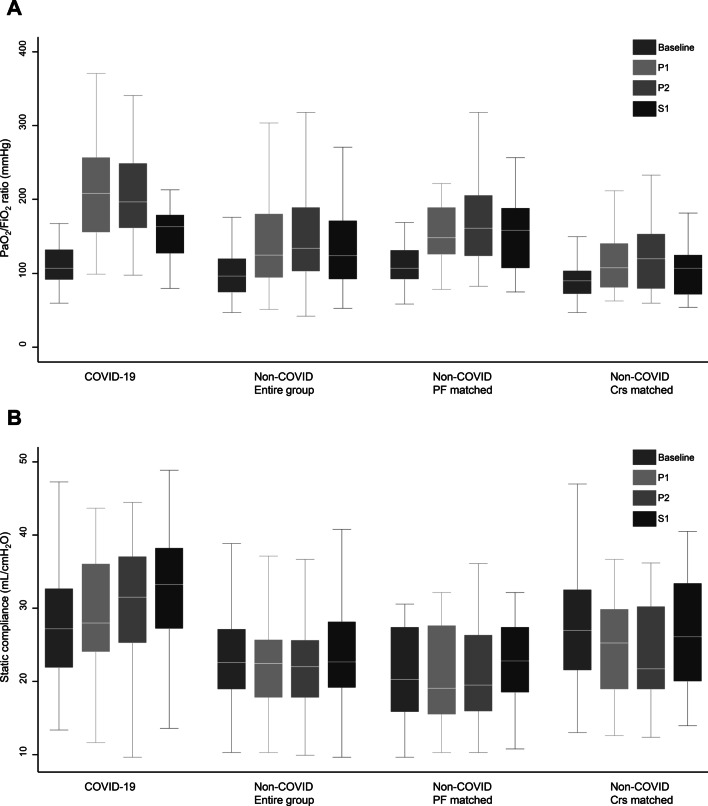
Changes in PaO_2_/FiO_2_ ratio and static respiratory system compliance after the first session of prone positioning. **A** PaO_2_/FiO_2_ ratio; **B** Static respiratory system compliance

The changes in PaO_2_/FiO_2_ ratio and static Crs after the first session of prone positioning are described in Fig. 1 and Additional file 1: Table S1. Baseline measurements were performed at a median of 1.3 (IQR 0.7–2.8) hours before initiation of prone positioning. Measurements for P1 and P2 timepoints were performed at a median of 9.7 (IQR 7.6–11.2) hours and 16.0 (IQR 13.5–17.8) hours after initiation of prone positioning, respectively. Most patients with COVID-19 ARDS showed improvement in both PaO_2_/FiO_2_ ratio and static Crs after prone positioning. The increase in PaO_2_/FiO_2_ ratio was the most prominent at the P1 timepoint and it slightly decreased when patients were moved to a supine position (Fig. 1A and Additional file 1: Table S1). The static Crs showed a continuous gradual increase during the first prone positioning session (Fig. 1B and Additional file 1: Table S1). A detailed comparison between patients with COVID-19 ARDS and those with non-COVID ARDS is presented in Table 3.

**Table 3:** PaO_2_/FiO_2_ ratio and static respiratory system compliance responses after the first prone positioning session

	Entire group		PaO_2_/FiO_2_-matched		Compliance-matched	
	N = 168	*P* value	N = 46	*P* value	N = 46	*P* value
PaO_2_/FiO_2_ ratio						
Change at the end of the first prone positioning session, mmHg						
COVID-19 ARDS, median (IQR)	89.8 (35.3–135.2)	0.003	89.8 (35.3–135.2)	0.091	89.8 (35.3–135.2)	< 0.001
Non-COVID ARDS, median (IQR)	40.1 (7.0–77.5)		48.7 (5.0–87.0)		21.1 (0.0–43.8)	
Regression coefficient (COVID vs. non-COVID)						
Unadjusted (95% CI)	46.7 (17.8–75.6)	0.002	32.5 (− 14.7–79.8)	0.172	69.7 (31.2–108.2)	0.001
Adjusted (95% CI)^a^	39.3 (5.2–73.5)	0.024	4.0 (− 62.9–71.0)	0.903	58.4 (− 13.5–130.2)	0.108
Static respiratory system compliance						
Change at the end of the first prone positioning session, mL/cmH_2_O						
COVID-19 ARDS, median (IQR)	3.7 (− 1.0–7.3)	< 0.001	3.7 (− 1.0–7.3)	0.044	3.7 (− 1.0–7.3)	< 0.001
Non-COVID ARDS, median (IQR)	− 0.3 (− 4.0–1.9)		0.4 (− 1.2–2.3)		− 2.3 (− 7.2–0.0)	
Regression coefficient (COVID vs. non-COVID)						
Unadjusted (95% CI)	4.2 (2.3–6.2)	< 0.001	2.7 (0.0–5.5)	0.047	6.6 (3.8–9.3)	< 0.001
Adjusted (95% CI)^a^	3.4 (1.1–5.6)	0.003	0.3 (− 3.3–3.8)	0.883	2.2 (− 2.4–6.7)	0.340

*CI* confidence interval, *FiO*_*2*_ fraction of inspired oxygen, *IQR* interquartile range, *PaO*_*2*_ partial pressure of oxygen

^a^For multivariable linear regression, the following variables were adjusted: age, sex, body mass index, duration of mechanical ventilation before initiation of prone positioning, sequential organ failure assessment (SOFA) score, Charlson comorbidity index (CCI), baseline setting of mechanical ventilator (positive end-expiratory pressure and tidal volume), and baseline respiratory mechanics before initiation of prone positioning (PaO_2_/FiO_2_, static compliance, and ventilatory ratio)

When comparing baseline and P2 timepoints, the absolute improvement in PaO_2_/FiO_2_ ratio was higher in patients with COVID-19 ARDS [median 89.8 (IQR 35.3–135.2) mmHg] than in patients with non-COVID ARDS [median 40.1 (IQR 7.0–77.5) mmHg]. The difference between the two groups remained significant after adjusting for other variables when the analysis was conducted using the entire group of patients with non-COVID ARDS [adjusted difference 39.3 (95% CI 5.2–73.5) mmHg]. However, when compared with the matched subgroups of patients with non-COVID ARDS, it was not significant [adjusted difference 4.0 (95% CI − 62.9–71.0) mmHg in PaO_2_/FiO_2_-matched samples and 58.4 (95% CI − 13.5–130.2) mmHg in compliance-matched samples].

The absolute change in static Crs between baseline and P2 timepoints was also higher in patients with COVID-19 ARDS [median 3.7 (IQR − 1.0–7.3) mL/cmH_2_O] than in patients with non-COVID ARDS [median − 0.3 (IQR − 4.0–1.9) mL/cmH_2_O]. However, similar to the case of PaO_2_/FiO_2_ ratio, after adjusting for other variables, this difference in static Crs was significant only when the analysis was conducted using the entire group of patients with non-COVID ARDS [adjusted difference 3.4 (95% CI 1.1–5.6) mL/cmH_2_O]. The significance was lost in the analysis of matched samples [adjusted difference 0.3 (95% CI − 3.3–3.8) mL/cmH_2_O in PaO_2_/FiO_2_-matched samples and 2.2 (95% CI − 2.4–6.7) mL/cmH_2_O in compliance-matched samples].

As a sensitivity analysis, we compared the relative percentage changes in PaO_2_/FiO_2_ ratio and static Crs between the two groups (COVID-19 ARDS and non-COVID ARDS). The relative percentage change was calculated as the absolute change divided by the baseline reference value. The results of this sensitivity analysis were similar to those of the main analysis (Additional file 1: Table S2). The unmatched analysis suggested that the relative percentage improvement in PaO_2_/FiO_2_ ratio and static Crs was more prominent in patients with COVID-19 ARDS than in patients with non-COVID ARDS. However, the significance was lost in the analysis of matched samples.

Given that the respiratory distress in patients with non-COVID ARDS had various underlying causes, we performed a subgroup analysis according to the etiology of ARDS in patients with non-COVID ARDS. Of 145 patients with non-COVID ARDS, pneumonia was the leading cause of ARDS in 124 patients (85.5%). We compared patients by dividing them into three groups: COVID-19 ARDS, non-COVID ARDS due to pneumonia, and non-COVID ARDS not due to pneumonia (Additional file 1: Table S3). Among the patients with non-COVID ARDS, oxygenation and Crs responses to prone positioning did not differ regardless of whether the underlying cause was pneumonia or not.

### Association between clinical outcomes

In patients with COVID-19 ARDS, successful discontinuation of mechanical ventilation was achieved in 16 of 23 patients (69.6%) within 90 days after the first session of prone positioning. We evaluated whether the changes in PaO_2_/FiO_2_ ratio and static Crs after the first prone positioning session were associated with successful discontinuation of mechanical ventilation. Improvement in PaO_2_/FiO_2_ ratio was more pronounced in patients who successfully discontinued mechanical ventilation than in those who did not [median 112 (IQR 85–144) vs. 35 (IQR 6–52) mmHg, *P* = 0.003]. In patients who successfully discontinued mechanical ventilation, static Crs increased by a median of 5.7 (IQR 3.3–7.7) mL/cmH_2_O, whereas in patients who did not, it decreased by a median of 1.0 (IQR − 3.0–3.7) mL/cmH_2_O (*P* = 0.006).

**Fig. 2:**
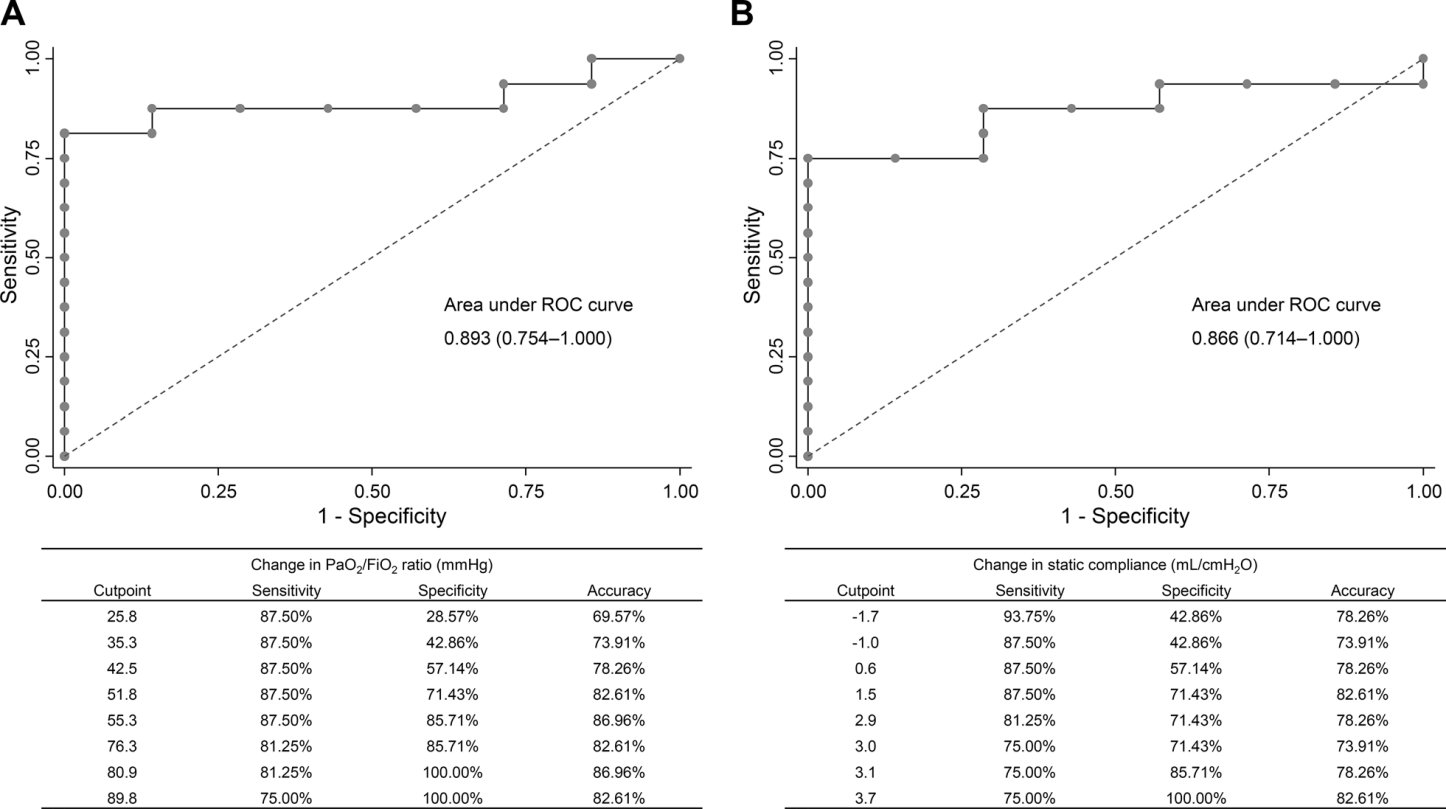
Receiver operating characteristic curve for changes in PaO_2_/FiO_2_ ratio and static respiratory system compliance in predicting the successful discontinuation of mechanical ventilation. **A** PaO_2_/FiO_2_ ratio; **B** Static respiratory system compliance

In ROC analysis, the areas under the curve were 0.893 (0.754–1.000) for the change in PaO_2_/FiO_2_ ratio and 0.866 (0.714–1.000) for the change in static Crs in predicting successful discontinuation of mechanical ventilation within 90 days (Fig. 2). In competing risk regression analysis, the extent of improvement in PaO_2_/FiO_2_ ratio (SHR 1.19, 95% CI 1.08–1.30 per 10 mmHg increase) and static Crs (SHR 1.57, 95% CI 1.29–1.91 per 1 mL/cmH_2_O increase) after the first prone positioning session were both associated with successful discontinuation of mechanical ventilation (Table 4). Among other variables, female sex, lower SOFA score, and higher baseline static Crs were associated with higher probability of successful discontinuation of mechanical ventilation.

**Table 4:** Predictors of successful discontinuation of mechanical ventilation for patients with COVID-19 ARDS

Predictors (N = 23)	Subdistribution hazard ratio^a^	*P* value
Age (per 1 year)	1.18 (0.99–1.40)	0.063
Female sex (vs. Male sex)	13.92 (1.17–165.15)	0.037
SOFA score (per 1 point)	0.68 (0.49–0.95)	0.022
Charlson comorbidity index (per 1 point)	0.48 (0.17–1.39)	0.176
Baseline PaO_2_/FiO_2_ ratio (per 10 mmHg)	0.73 (0.53–1.00)	0.054
Baseline static respiratory system compliance (per 1 mL/cmH_2_O)	1.40 (1.10–1.79)	0.006
Increase in PaO_2_/FiO_2_ ratio after the first prone positioning session (per 10 mmHg)	1.19 (1.08–1.30)	< 0.001
Increase in static respiratory system compliance after the first prone positioning session (per 1 mL/cmH_2_O)	1.57 (1.29–1.91)	< 0.001

*FiO*_*2*_ fraction of inspired oxygen, *PaO*_*2*_ partial pressure of oxygen, *SOFA* sequential organ failure assessment

^a^Subdistribution hazard ratios are described with their 95% confidence intervals

### Literature review for related studies

Given the limited sample size of our study, we performed additional literature review for other related studies investigating the physiological effects of prone positioning in mechanically ventilated patients with COVID-19 ARDS (Table 5). As of June 2021, we were able to identify 16 studies, and all studies retrieved showed that prone positioning substantially improves oxygenation in patients with COVID-19 ARDS. However, responses of static Crs varied between the studies.

**Table 5:** Literature review for studies evaluating efficacy of prone positioning in mechanically ventilated patients with COVID-19 ARDS

Study author	Number of patients	Study region	Timing of response evaluation	Change in PaO_2_/FiO_2_ ratio (mmHg)	Change in compliance (mL/cmH_2_O)
Present study by Jimyung Park	23	South Korea	End of first proning session	Median 107 (IQR 92–132) → median 196 (IQR 161–248)	Median 27.2 (IQR 21.9–32.7) → median 31.5 (IQR 25.2–37.0)
Osama Abou-Arab [41]	25	France	End of first proning session	Median 91 (95% CI 78–137) → median 124 (95% CI 97–149)	Median 32 (95% CI 21–38) → median 32 (95% CI 23–40)
Alfredo J Astua [42]	29	U.S.A	End of first proning session	Mean 107.5 ± 5.6 → mean 142.0 ± 10.8	N.A
Max Berrill [43]	34	U.K	End of every proning session	Mean 99.8 ± 100 → mean 151.9 ± 58.9	N.A
Jennifer Clarke [44]	20	Ireland	During first proning session	Median 123 (IQR 100–154) → median 286 (IQR 195–348)	Median 33.7 (IQR 30.1–43.0) → median 32.5 (IQR 26.7–37.5)
Ivor S Douglas [45]	61	U.S.A	2 h after starting proning	Median 99 (IQR 73–128) → median 136 (IQR 105–164)	N.A
Helena Gleissman [46]	44	Sweden	End of first proning session	Median 104 (IQR 86–122) → median 161 (IQR 127–207)	N.A
Rohit Khullar [47]	23	U.S.A	End of last proning session	Mean 84.8 (SD N.A.) → mean 202.0 (SD N.A.)	N.A
Thomas Langer [48]	78	Italy	End of first proning session	Median 98 (IQR 72–121) → median 158 (IQR 112–220)	Median 43 (IQR 31–50) → median 42 (IQR 35–48)
Mirja Mittermaier [49]	9	Germany	12 h after starting proning	Mean 118.4 ± 41.9 → mean 181.8 ± 63.2	N.A
François Perier [50]	9	France	3 h after starting proning	N.A	Median 44 (IQR 38–55) → median 39 (IQR 32–53)
Ling Sang [51]	20	China	End of first proning session	Mean 68.0 ± 10.3 → mean 82.4 ± 15.5	Mean 17.5 ± 3.5 → mean 20.6 ± 4.4
Gaetano Scaramuzzo [52]	191	Italy	3 h after resupination	Median 49% improvement (IQR 19–100%)	N.A
Mehdi C Shelhamer [53]	62	U.S.A	During first proning session	Improvement by 36.4 mmHg (49% improvement)	N.A
Richard Vollenberg [54]	13	Germany	6 h after starting proning	Median 58% improvement (IQR 31–95%)	Median 38 (IQR 26–58) → median 39 (IQR 27–59)
Tyler T Weiss [29]	36	U.S.A	2 h after starting proning	Median 131 (IQR 87–144) → median 208 (IQR 146–268)	Median 29.2 (IQR 23.3–35.5) → median 29.2 (IQR 24.0–36.2)
David R Ziehr [55]	122	U.S.A	End of first proning session	Median 149 (IQR 123–170) → median 235 (IQR 186–285)	Median 31 (IQR 27–39) → median 33 (IQR 28–38)

*CI* confidence interval, *FiO*_*2*_ fraction of inspired oxygen, *IQR* interquartile range, *N.A.* not available, *PaO*_*2*_ partial pressure of oxygen, *SD* standard deviation

## Discussion

In this study, we compared the physiological response of prone positioning between patients with COVID-19 ARDS and non-COVID ARDS, focusing on changes in oxygenation and static Crs. Most patients with COVID-19 ARDS showed improvement in PaO_2_/FiO_2_ ratio and static Crs after the first session of prone positioning. The extent of improvement in these parameters appeared to be higher in patients with COVID-19 ARDS when compared crudely with the entire group of patients with non-COVID ARDS. However, when 1:1 matched samples (PaO_2_/FiO_2_-matched and compliance-matched) were analyzed, the physiological response to prone positioning was not different between patients with COVID-19 ARDS and those with non-COVID ARDS.

Whether patients with COVID-19 ARDS have a clinically different phenotype compared with those with typical non-COVID ARDS continues to be a controversial issue [5, 22]. One of the issues related to this controversy is regarding static Crs. Since the COVID-19 pandemic started, some patients with COVID-19 ARDS have been reported to have preserved static Crs despite impaired oxygenation, which is referred to as “type L (low elastance) phenotype” compared with “type H (high elastance) phenotype” [22, 23]. A multicenter study in Italy reported that patients with COVID-19 ARDS had higher median static Crs than those with non-COVID ARDS (41 vs. 32 mL/cmH_2_O), although there was a substantial overlap between the two groups [11]. However, in several other studies, patients with COVID-19 ARDS presented with static Crs of approximately 30–35 mL/cmH_2_O, which is similar to that in previous reports of typical non-COVID ARDS [6, 10, 24–27].

In our study, patients in both groups showed substantially reduced static Crs (median 27.2 and 21.9 mL/cmH_2_O in COVID-19 and non-COVID group, respectively). Especially, patients with non-COVID ARDS in this study had extremely poor static Crs considering that a recent secondary analysis of the LUNG SAFE study, which included a large multinational cohort of patients, reported the median static Crs of 30 mL/cmH_2_O [28]. This may be due to the selection bias that occurs in single-center studies. In fact, we could not identify any patient in either group (COVID-19 or non-COVID) who can be classified as having type L phenotype (static Crs ≥ 50 mL/cmH_2_O). Therefore, our findings may not be applicable to patients with type L phenotype.

Almost every patient with COVID-19 ARDS in this study showed improvement in PaO_2_/FiO_2_ ratio after prone positioning. Such improvement was rapid and most noticeable after 10 h of prone positioning. This finding is consistent with that of another single-center study of intubated patients with COVID-19 treated using prone positioning, which reported that PaO_2_/FiO_2_ ratio improved within 2 h after initiation of prone positioning [29]. In a prospective study of prone positioning in nonintubated patients, improvement in oxygenation was observed even 10 min after initiation of prone positioning [30]. In contrast, a previous study on non-COVID ARDS showed that the oxygenation status was not always improved immediately after initiation of prone positioning [31]. In other studies, including the PROSEVA trial, PaO_2_/FiO_2_ ratio was higher at the end of the prone positioning session than at 1 h after initiation of prone positioning, which is similar to our findings for patients with non-COVID ARDS [7, 32]. Based on these findings, it can be suggested that the speed of the oxygenation response after prone positioning may differ between patients with COVID-19 ARDS and those with non-COVID ARDS. Because PaO_2_/FiO_2_ ratio cannot be monitored on real-time basis, monitoring oxygenation based on SpO_2_/FiO_2_ ratio might provide more information on this issue.

The change in static Crs after prone positioning has not been studied as much as the change in oxygenation. In one study, static Crs was improved with prone positioning when it was accompanied only with application of high PEEP, but not with low PEEP [33]. Crs is determined by compliance of the chest wall and lung. Because chest wall compliance usually decreases during prone positioning, the overall change in Crs after prone positioning depends on how much the compliance of the lung improves, which may be related to lung recruitability [8]. In our study, the extent of improvement in static Crs after prone positioning appeared to be higher in patients with COVID-19 ARDS than in patients with non-COVID ARDS in a crude analysis. However, the difference was not significant when the analysis was performed using the matched samples. In addition to static Crs, it may be useful to monitor the lung recruitability while implementing prone positioning [34–37].

The major finding of our study was that oxygenation and Crs responses after prone positioning were not different between patients with COVID-19 ARDS and those with non-COVID ARDS after careful matching and adjusting for baseline between-group differences. It is intriguing that the unmatched analysis suggested that prone positioning was more effective in patients with COVID-19 ARDS than in those with non-COVID ARDS. However, this finding may have resulted from the effects of unmeasured confounding factors, suggesting that our 1:1 matched analysis is more appropriate for a proper comparison. Taking the findings of both unmatched and 1:1 matched analyses into account, the physiological effects of prone positioning in COVID-19 ARDS may be comparable with, or at least not inferior to, those in typical non-COVID ARDS.

In fact, because non-COVID ARDS comprises lung injuries from very heterogeneous causes, it is not easy to make a proper comparison between the two groups. Furthermore, although COVID-19 ARDS occurs by infection caused by a common single pathogen, results of several studies indicated that respiratory mechanics of patients with COVID-19 ARDS show a substantial interindividual variability, highlighting the importance of individualization in ventilator management [38]. As in our study, it may be because of this interindividual variability that other studies also failed to identify significant differences between COVID-19 ARDS and non-COVID ARDS [39, 40].

We have recently reported that the extent of improvement in oxygenation after the first session of prone positioning could be predictive of clinical outcome for patients with non-COVID ARDS [9]. In this study, we confirmed this finding in patients with COVID-19 ARDS. In addition, we found that the improvement in static Crs after prone positioning was also associated with clinical outcome. Our findings suggest that if the physiological effect of prone positioning is not substantial at the end of the first session, intensivists may have to consider other therapeutic options. By comparison, a post hoc analysis of the PROSEVA trial found no association between the improvement in oxygenation after 1 h of prone positioning and survival outcomes [16]. This discrepancy may have arisen from the difference in the timing of evaluating the response to prone positioning. Given that it is not clear which timepoint after initiating prone positioning is most appropriate for response evaluation, more studies are needed to clarify this issue.

Our study has several limitations. First, our study was conducted at a single center and the number of patients studied was limited, although we enrolled every consecutive patient treated using prone positioning until December 2020. To compensate for this limitation, we performed additional literature review for other related studies. All studies retrieved consistently showed that prone positioning is effective in improving oxygenation in patients with COVID-19 ARDS. Second, despite our efforts to adjust for between-group differences including 1:1 matched analysis, we cannot exclude the possibility that uncontrolled individual factors affected our study findings. Third, we could not evaluate the effect of prone positioning in patients with preserved static Crs (type L phenotype), because there were no such patients in our cohort.

## Conclusions

In conclusion, in patients with COVID-19 ARDS, prone positioning was as effective in improving oxygenation and static Crs as in patients with non-COVID ARDS. Although interindividual variability in respiratory mechanics indicates the need for more individualized approaches in ventilator management, our study findings suggest that prone positioning should be actively considered for patients with moderate-to-severe COVID-19 ARDS. In addition, the physiological response to the first session of prone positioning should be monitored to predict the future clinical outcome.

## Supplementary Information

**Additional file 1:Table S1.** Change in PaO2/FiO2 ratio and static respiratory system compliance after prone positioning; **Table S2.** Relative percentage change in PaO2/FiO2 ratio and static respiratory system compliance; **Table S3.** Subgroup analysis according to underlying cause of non-COVID ARDS.

## Data Availability

The datasets used and/or analyzed during the current study are available from the corresponding author on reasonable request.

## Abbreviations

ARDS: Acute respiratory distress syndrome
CCI: Charlson comorbidity index
CI: Confidence interval
COVID-19: Coronavirus disease 2019
FiO_2_: Fraction of inspired oxygen
IQR: Interquartile range
PaO_2_: Partial pressure of arterial oxygen
PEEP: Positive end-expiratory pressure
ROC: Receiver operating characteristics
Crs: Respiratory system compliance
SOFA: Sequential organ failure assessment
SHR: Subdistribution hazard ratio
