# Supplementary material for: Effect of prone positioning on oxygenation and static respiratory system compliance in COVID-19 ARDS vs. non-COVID ARDS
Source: Respir Res. 2021 Aug 6;22:220. doi: 10.1186/s12931-021-01819-4 (PMC8343350; doi:10.1186/s12931-021-01819-4)
Supplement: Supplementary file 1 — Additional file 1:Table S1. Change in PaO2/FiO2 ratio and static respiratory system compliance after prone positioning; Table S2. Relative percentage change in PaO2/FiO2 ratio and static respiratory system compliance; Table S3. Subgroup analysis according to underlying cause of non-COVID ARDS. [file 12931_2021_1819_MOESM1_ESM.docx]

**Additional file 1**

**Effect of prone positioning on oxygenation and static respiratory system compliance in COVID-19 ARDS versus non-COVID ARDS**

Jimyung Park, Hong Yeul Lee, Jinwoo Lee, Sang-Min Lee

Division of Pulmonary and Critical Care Medicine, Department of Internal Medicine, Seoul National University Hospital, Seoul, Republic of Korea

**Corresponding author**: Sang-Min Lee, M.D.

Division of Pulmonary and Critical Care Medicine, Department of Internal Medicine, Seoul National University College of Medicine, 101, Daehak-ro, Jongno-gu, Seoul, 03080, Republic of Korea

Telephone: +82-2-2072-0833, Fax: +82-2-762-9662, E-mail: [sangmin2@snu.ac.kr](mailto:sangmin2@snu.ac.kr)

**Table S1. Change in PaO_2_/FiO_2_ ratio and static respiratory system compliance after prone positioning**

|  | COVID-19 ARDS | | | Non-COVID ARDS | | |
| --- | --- | --- | --- | --- | --- | --- |
|  | Value  (median) | Difference  (95% CI) | *P* value | Value | Difference | *P* value |
| PaO_2_/FiO_2_ ratio, mmHg | | | | | | |
| Baseline | 107 (92–132) | Reference |  | 96 (74–120) | Reference |  |
| P1 | 208 (156–257) | 102 (70–134) | <0.001 | 125 (95–180) | 43 (34–52) | <0.001 |
| P2 | 196 (161–248) | 97 (62–133) | <0.001 | 134 (102–189) | 51 (40–61) | <0.001 |
| S1 | 163 (128–179) | 44 (27–62) | <0.001 | 124 (93–171) | 35 (26–44) | <0.001 |
|  |  |  |  |  |  |  |
| Static compliance, mL/cmH_2_O | | | | | | |
| Baseline | 27.2 (21.9–32.7) | Reference |  | 21.9 (18.2–26.5) | Reference |  |
| P1 | 27.9 (24.0–36.0) | 1.7 (-0.4–3.8) | 0.073 | 21.7 (17.0–25.1) | -1.0 (-1.7–-0.3) | 0.016 |
| P2 | 31.5 (25.2–37.0) | 3.4 (1.3–5.4) | 0.006 | 21.3 (17.0–25.0) | -0.9 (-1.6–-0.2) | 0.057 |
| S1 | 33.2 (27.2–38.2) | 5.3 (2.8–7.7) | <0.001 | 22.0 (18.4–27.6) | 0.9 (-0.2–2.0) | 0.208 |

ARDS, acute respiratory distress syndrome; FiO_2_, fraction of inspired oxygen; PaO_2_, partial pressure of oxygen

PaO_2_/FiO_2_ ratio and static compliance were evaluated at four timepoints for each patient: baseline (before initiation of prone positioning), P1 (approximately 10 hours after initiation of prone positioning), P2 (approximately 16 hours after initiation of prone positioning, which is the last timepoint before cessation of prone positioning), and S1 (approximately 2 hours after changing to supine position).

*P* values are for intra-individual comparison between different timepoints, calculated by the Wilcoxon signed-rank test using baseline values as a reference.

**Table S2. Relative percentage change in PaO_2_/FiO_2_ ratio and static respiratory system compliance**

|  | Entire group |  | PaO_2_/FiO_2_-matched |  | Compliance-matched |  |
| --- | --- | --- | --- | --- | --- | --- |
|  | N = 168 | *P* value | N = 46 | *P* value | N = 46 | *P* value |
| PaO_2_/FiO_2_ ratio |  |  |  |  |  |  |
| Relative percentage change at the end of the first  prone positioning session, %  COVID-19 ARDS, median (IQR)  Non-COVID ARDS, median (IQR) | 81 (34–116)  42 (7–84) | 0.028 | 81 (34–116)  40 (5–83) | 0.106 | 81 (34–116)  30 (0–51) | 0.006 |
| Regression coefficient (COVID vs. non-COVID)  Unadjusted (95% CI)  Adjusted (95% CI)^a^ | 39 (-1–78)  40 (-4–85) | 0.051  0.075 | 34 (-19–87)  5 (-65–75) | 0.205  0.885 | 63 (17–109)  67 (-14–148) | 0.008  0.104 |
|  |  |  |  |  |  |  |
| Static respiratory system compliance |  |  |  |  |  |  |
| Relative percentage change at the end of the first  prone positioning session, %  COVID-19 ARDS, median (IQR)  Non-COVID ARDS, median (IQR) | 16 (-4–24)  -2 (-16–11) | 0.001 | 16 (-4–24)  3 (-7–17) | 0.060 | 16 (-4–24)  -12 (-23–0) | <0.001 |
| Regression coefficient (COVID vs. non-COVID)  Unadjusted (95% CI)  Adjusted (95% CI)^a^ | 15 (6–23)  11 (2–21) | 0.001  0.018 | 10 (-1–21)  3 (-12–17) | 0.071  0.710 | 23 (12–33)  5 (-13–24) | <0.001  0.562 |

CI, confidence interval; FiO_2_, fraction of inspired oxygen; IQR, interquartile range; PaO_2_, partial pressure of oxygen

^a^ For multivariable linear regression, the following variables were adjusted: age, sex, body mass index, duration of mechanical ventilation before initiation of prone positioning, sequential organ failure assessment (SOFA) score, Charlson comorbidity index (CCI), baseline setting of mechanical ventilator (positive end-expiratory pressure and tidal volume), and baseline respiratory mechanics before initiation of prone positioning (PaO_2_/FiO_2_, static compliance, and ventilatory ratio).

**Table S3. Subgroup analysis according to underlying cause of non-COVID ARDS**

|  | COVID-19  ARDS | Non-COVID  ARDS due to pneumonia | Non-COVID  ARDS not due to pneumonia |
| --- | --- | --- | --- |
|  | N = 23 | N = 124 | N = 21 |
| Age, years | 70 (63–74) | 68 (60–74) | 66 (40–73) |
| Male sex | 15 (65.2%) | 83 (66.9%) | 14 (66.7%) |
| Body mass index, kg/m^2^ | 25.6 (22.9–27.4) | 22.8 (20.6–25.9) | 23.7 (21.5–26.1) |
| Charlson comorbidity index | 4 (3–4) | 5 (4–8) | 3 (1–5) |
| APACHE II score | 20 (12–25) | 30 (25–34) | 28 (23–33) |
| SAPS II score | 42 (31–61) | 66 (56–72) | 56 (46–65) |
| SOFA score | 8 (5–11) | 12 (9–14) | 11 (10–13) |
| Ventilator FiO_2_ | 0.7 (0.6–0.8) | 0.8 (0.6–1.0) | 0.9 (0.6–1.0) |
| PaO_2_/FiO_2_ ratio, mmHg | 107 (92–132) | 96 (74–120) | 102 (64–108) |
| PEEP, cmH_2_O | 12 (9–13) | 10 (8–11) | 8 (8–10) |
| Driving pressure, cmH_2_O | 13 (12–16) | 18 (15–20) | 20 (18–22) |
| Respiratory rate, breaths/min | 21 (19–27) | 27 (24–30) | 26 (25–32) |
| Static compliance, mL/cmH_2_O | 27.2 (21.9–32.7) | 22.2 (18.2–27.1) | 20.9 (18.2–24.8) |
| Ventilatory ratio | 1.7 (1.4–2.0) | 2.2 (1.7–2.7) | 2.2 (2.1–3.0) |
| Change in PaO_2_/FiO_2_ ratio, mmHg  Value  Unadjusted difference  Adjusted difference^a^ | 89.8 (35.3–135.2)  44.7 (15.4–73.9)  38.0 (3.4–72.5) | 40.0 (7.8–79.2)  Reference  Reference | 43.8 (2.5–58.8)  -14.2 (-44.6–16.1)  -9.4 (-41.6–22.8) |
| Change in static compliance, mL/cmH_2_O  Value  Unadjusted difference  Adjusted difference^a^ | 3.7 (-1.0–7.3)  4.2 (2.2–6.2)  3.2 (1.0–5.5) | 0 (-4.0–2.2)  Reference  Reference | -1.0 (-3.9–0.4)  -0.5 (-2.6–1.6)  -0.9 (-3.0–1.2) |

APACHE, Acute Physiology and Chronic Health Evaluation; ARDS, acute respiratory distress syndrome; FiO_2_, fraction of inspired oxygen; PaO_2_, partial pressure of oxygen; PEEP, positive end expiratory pressure; SAPS, Simplified Acute Physiology Score; SOFA, Sequential Organ Failure Assessment

^a^ For multivariable linear regression, the following variables were adjusted: age, sex, body mass index, duration of mechanical ventilation before initiation of prone positioning, sequential organ failure assessment (SOFA) score, Charlson comorbidity index (CCI), baseline setting of mechanical ventilator (positive end-expiratory pressure and tidal volume), and baseline respiratory mechanics before initiation of prone positioning (PaO_2_/FiO_2_, static compliance, and ventilatory ratio).
